# The Invalid Children's Aid Association

**Published:** 1905-05-27

**Authors:** 


### THE INVALID CHILDREN'S AID ASSOCIATION.

### SOME CHEERING SPEECHES.

Lord Midleton presided at the annual meeting of the Invalid Children's Aid Association, held at Bolney House, Ennismore Gardens, on Wednesday the 17th instant. There was a large attendance.

In opening the proceedings, the Chairman said that perusal of the very lucid report for the past year provided conclusive proof that there was a definite want to be supplied, and that the means employed were adequate and successful. No one who knew the condition of the poorer classes could doubt the want of constant surveillance over the feeble and afflicted if they were to obtain proper treatment. It was so in the country, but more emphatically in the crowded hives of industry, owing to the abnormal conditions of life of the great mass of the people. Going about London he had come across houses, once perhaps homes of wealth, now let in tenements, sublet by floors, again sublet by rooms, and sometimes even the rooms sublet to families, one in each corner and perhaps one in the centre. Even where things were not quite so bad as that, when sickness, and especially lingering sickness, invaded what was called the home, it was necessary to deal with it in the complete absence of all the appliances and conditions necessary for successful treatment. This Association was dealing with 4,000 cases a year at a cost of about £5,000, which worked out at about 25s. per case, so that it could not be blamed with undue extravagance. This keeping down of expenses was due to the large amount of voluntary work; work of the best kind, done without fee or reward—often, indeed, without appreciation, and with very scant knowledge on the part of the public. The result was that a large number of sick and crippled children, who were spending their lives under conditions precluding the possibility of a return to health, were helped to get fresh air, better food, and proper appliances, and were taught habits of cleanliness and hygiene which could not but be of the greatest benefit in after life. There were 56 different centres from which this blessed work was carried on. The voluntary visitors were assisted by the paid agents of the Association where necessary; there was complete co-operation with other agencies, and it seemed only necessary to make the public acquainted with the work that was being done for it to enjoy a much greater measure of support. It was an effort on the part of the leisured classes to enter into the cheerless lives of those less favourably situated, to care something for others, and to extend the helping hand to those in need, so that, in the words of John Bright, "there may be no more complaining in our streets."

### Excellent and Good Work.

Sir Henry Burdett moved the adoption of the report and the re-election of the committee and council. He wished, he said, to appeal to the individual citizen to do what each one could in a work like that of this society, which was essential to the happiness and the health of so many otherwise helpless children. He would remind the well-to-do of that vast city that they could not live in comfort and in health unless they had in their midst an ever-increasing army of those whose business it was to do the rough work necessary to secure the well-being of every class. The poor had a very hard time, but they had their rights; and it was the privilege of the rich not only to recognise those rights, but to take the trouble to see that the poorer people got them. Therefore, that Association was one which ought to have the support and grateful recognition of every one in London. Its aim was to go into the byways and streets and to dig out and save the children of the poor. The great difficulty in so vast a population was to get at the truth, and get at the individual. That Association did both, and had lessened the suffering in thousands of homes. It had restored to health many children who otherwise would have been crippled for life, even if they had contrived to live at all. The work was well organised, and unlike some organisations, it was not in rivalry or competition with others but stood alone. It acted in co-operation with every organisation which was in touch with the homes and the children of the poor. It dealt with the patients at the hospitals, looking after the cases, and saw that they got what the hospitals could not give, fresh air, and further treatment when they left them. It co-operated with medical men, with district and other nursing associations, with school authorities, the Charity Organisation Society, with the clergy and all connected with religious agencies, and with the Ragged School Union; and in addition, so much good had been done and so high was the opinion it had gained, that it had the love and affection of the children and the parents themselves. Theirs was a glorious army, and he, as a worker in London, felt his heart go out to every one of the 365 workers, going up and down and in and out of the homes of the poor to find out and recover many suffering children who would otherwise be lost. Surely such an Association was one with which every thinking man and woman ought to be connected and in some way to support. It was a great thing that everything was done to provide that immediately a need was discovered it should be promptly remedied. What other Association could say that it had a body of ladies and gentlemen meeting regularly twice a week in order that the wants of those in their care might be promptly attended to? There was a thoroughness about the work that was highly commendable. After medical aid had been given, the child was followed back to the family, arrangements were made for it to go into a convalescent or some cottage home, and afterwards it was looked after till it was 15 years old, and care was taken that it should be attached to some business or trade so that its livelihood should be secured. These were special features of the work that were as commendable as they were remarkable, and represented the combination of philanthropy and common sense that distinguished the Association. Of course, in work of that kind, though the progress had been great in the 17 years of the Society's history, yet it had not been enough to overtake the number of cases to be dealt with. As the work increased the need for cash and for workers increased with it. At present they were able to do for each child at an average cost of something like 25s. It was always good to have a certain sum to put before people, so that they might know directly what was the unit of expenditure. He hoped that everybody would get out of the habit of giving the customary guinea and increase it to 25s. a year at least, and so each provide the cost of a case. He considered that in the days of health every man and woman owed it to God to render a measure of personal service, and that Association provided a splendid opportunity. No one need hold back for lack of knowledge, since when they began they would be assigned to a district to work under an experienced person who would indoctrinate the novices and show them how to do the work efficiently. Therefore, he said to everyone, do not hesitate to begin, since even a child can help a child and so have an opportunity of beginning a career of usefulness and service. Let him give them a case that had come under his own notice which well illustrated how they could help in saving life. There was a poor family in the Borough where a little boy had been kept on an old sofa for 12 years, and not allowed to walk; it was thought that he could not stand. One day a lady visiting in the neighbourhood heard of the case and went to see him. She interested her father and it was decided that the child should be examined medically. He was moved into Guy's Hospital, and put into the care of one of the surgeons who undertook the case and it was then found that the child was not as had been thought suffering from hereditary incapacity to walk but from a tumour which, if attended to before, would have saved years of weakness and weariness. This was dealt with and after a long time and much anxiety the lad was sent out able to walk and he could now go about hale and well, and was able to help the family and become a good citizen. Was there anyone who would not yearn for an opportunity to do what that lady did in this case? And it was the glory of that society that it was helping to save such lives every year. Then the Association had the merit of not only supplying instruments but of having them made specially to fit, and the visitors took care that they were properly used until the need for them disappeared. The Bishop of Stepney had said that the real test of the greatness of a man was the strength of his compassion; the movement which lifts a man up and brings him down to the needs and sufferings of his brethren. It was good to give money, but it was better to be a visitor and they wanted many more workers of both sexes. Would not each Londoner, man or woman, do what he could in the days of health for the good of their poorer neighbours and their sick children through this beneficent Association?

### From Ugliness to Beauty.

Professor Howard Marsh seconded the motion. He was glad, he said, to have an opportunity of saying something on the surgical aspect of the question. The longer he lived the more convinced he was of the essential necessity of the work of that Association. It was said that the poor little gutter brats whom they looked after had no constitution, and that time and effort could be used to better purpose than on them. That he altogether denied. He quite agreed that they were pale and thin and weakly, partly starved and wholly neglected in appearance. But change the circumstances and all that altered very quickly. Just as a flower, a poor mean stunted thing as long as it was in barren ground behind a brick wall and quite untended, handsomely repaid being moved to good soil and given sunshine and attention; so if they took a child out of a dirty court with unwholesome surroundings, and gave it proper conditions, they would not know it in a fortnight. He had seen, again and again, a dirty little pig of a child, disagreeable, almost, one might say, repulsive, taken to the Children's Hospital, and a change worked that was a complete transformation; clean, its hair cut, sleeping happily, fattening out and with returning colour—becoming in fact a beautiful child. One enormous good that was done was to restore the child's power of resistance to disease. It was a great mistake to believe that the diseases which attack children were incurable; generally they were more easily cured than in grown-ups. Tuberculosis was an instance of this. It was absolutely and entirely wrong to think of it as a constitutional disease acquired by inheritance. It was a condition which resulted from the invasion of the system by a bacillus; children in bad surroundings had a greater liability to succumb to the invasion when attacked, and their business was to help them to acquire resistance. He frequently saw cases where the disease, even after it had been acquired, had been absolutely and entirely got rid of; but just as with fire, the earlier it was attacked the better the hope of dealing with it successfully. The society, too, did a great deal of good by educating people in the proper management of children, and in this connection especially he must express his admiration of the way in which the staff did its work; for that no praise could be too great or too hearty.

### Some Good Stories.

Mr. Pett Ridge made a humorously pathetic speech, into which he introduced several stories. The room in which the meeting was held was a very handsome and commodious one, many of the ladies present being beautifully dressed, and as showing that the claim of those in need upon those better off could be conveyed by implication as well as by being thrown at them, he recounted a conversation overheard in Farringdon Street between a couple of newsboys. One, a sharp little cripple, was counting his takings; the other, a rather bigger lad, stood eating walnuts. "I'm a ha'penny short," said the first. The other cracked a fresh nut. "I'm a ha'penny short," repeated the cripple. "Oh! don't keep harking on it," said the other, "What's it to do with me? You don't want to say I've got it?" "Well," said the cripple, "I don't know. Here am I a ha'penny short, and there are you a-eating nuts! What?" In the speaker's opinion the days of youth were not all they were cracked up to be. They were described by grown-up people as great fun, but to his mind a joke that one could only see after the lapse of 20 years was not the simplest and purest and best form of humour. It was a truism of course, but it was apt to be forgotten, that the children came into the world by no desire of their own; they were not consulted whether they would be Limehouse children or Mayfair children. But there was a good deal of human nature in them all, and if they could not display it about one thing they did about another. Two little girls he had heard of went to the Long Lane Board School, and on arrival the teacher considered that their features were not sufficiently distinct and sent them to get a wash. As they were polishing up after it one said to the other with a fine assumption of majesty and hauteur, "My face was a lot dirtier than yours." "Yes," was the reply, "you're older!" (Laughter.) The secretary told him that in that Association they kept a Golden Book in which certain people's names were put, and that reminded him of the Golden Book in which Abou ben Adhem wished his name to be written. Concluding he said, "I like to think that those who are making sacrifices for these little people will find their reward; and to remember that 'inasmuch as ye have done it unto one of the least of these'—you know the rest?"

Miss Lilian Braithwaite also spoke, and after a vote of thanks to the chairman for presiding and to Mrs. Henry Tate for the use of her house for the meeting, the proceedings terminated.

